# Performance characteristics of lipoprotein-associated phospholipase A2 activity assay on the Dimension Vista analyser and preliminary study of a healthy Italian population

**DOI:** 10.11613/BM.2017.030701

**Published:** 2017-08-28

**Authors:** Alessandro De Stefano, Liliana Mannucci, Renato Massoud, Sergio Bernardini, Claudio Cortese

**Affiliations:** Department of Experimental Medicine and Surgery, University of Rome Tor Vergata, Rome, Italy

**Keywords:** Lp-PLA_2_, assay validation, cardiovascular disease, Darapladib, reference intervals

## Abstract

**Introduction:**

Lipoprotein-associated phospholipase A2 (Lp-PLA_2_) is a marker of vascular inflammation associated with coronary heart disease and stroke. We evaluated analytical performance of the PLAC® Activity Test on Siemens Dimension Vista® 1500 analyzer and measured Lp-PLA_2_ activity in Italian adults to establish reference intervals (RIs) and evaluate correlation with circulating lipids and age.

**Materials and methods:**

The evaluation protocol consisted of precision, linearity, sensitivity, method comparison, substrate depletion (“hook”) effect and interference assessment. Inhibitor (Darapladib) effect was also evaluated. Lp-PLA_2_ activity was measured in 250 healthy donors (123 males, 127 females, aged 18-70 years). Central 95% RIs were established using nonparametric statistics.

**Results:**

Intra-assay and inter-assay precision showed CVs of 0.6% - 1.4% and 0.9% - 2.0%, respectively. Linearity replicates showed R^2^ > 0.98. Limit of quantitation was 5.8 U/L (CV = 9.4%). Bland Altman plot showed bias - 0.9, 95% limits of agreement -6.5 - 4.72. Passing-Bablok regression showed excellent correlation (Slope = 1.02, 95% CI: 1.01 to 1.03; Intercept = - 1.86, 95% CI: - 3.08 to - 1.26; R^2^ = 0.999). No “hook effect” was observed at Lp-PLA_2_ activities ≤ 1000 U/L. Average Lp-PLA_2_ activity in 250 healthy donors was 182 ± 44 U/L (mean ± SD). Males showed statistically significant higher activities than females (P < 0.001). RIs were 107 - 265 U/L for males and 84 - 225 U/L for females. Moderate significant correlation (r = 0.29, P < 0.001) was found between Lp-PLA_2_ activity and total cholesterol.

**Conclusions:**

The PLAC® Activity Test shows very good performance characteristics on Dimension Vista® 1500.

## Introduction

Lipoprotein-associated phospholipase A2 (Lp-PLA_2_), also known as platelet-activating factor acetyl-hydrolase, is a calcium-independent serine lipase which was initially identified by its hydrolysing action on the acetyl group at the sn-2 position of platelet-activating factor (*1*). It is now known that Lp-PLA_2_ can hydrolyse a broad spectrum of substrates, including oxidized and polar phosphatidylcholines (*2*). Lp-PLA_2_ is synthesized by macrophages and other inflammatory cells and circulates in human blood mainly bound to low-density lipoprotein (LDL) particles (80 - 85%) and, to a lesser extent, to high-density lipoprotein (HDL) (*3*). Although it is considered an atheroprotective enzyme, the hydrolysis of oxidized phospholipids generates lysophospholipids, compounds that have a pro-inflammatory function (*4*). Several lines of evidence indicate that oxidized LDL play a critical role in atherogenesis and Lp-PLA_2_ has been shown to be involved in the oxidative modification of LDL in the vascular wall by hydrolysing oxidized phospholipids, generating lysophosphatidylcholine and oxidized non-esterified fatty acids, which are strong pro-inflammatory mediators contributing to the formation of the atherosclerotic plaque (*4*-*6*). Lp-PLA_2_ is a highly specific marker of vascular inflammation and its role in the development and progression of atherosclerosis has been suggested by various authors (*6*-*8*). High Lp-PLA_2_ activity during atherosclerotic plaque formation and other inflammatory processes have been shown in several studies and there is increasing evidence that this enzyme is present within atherosclerotic lesions and enriched in vulnerable regions (*6*, *8*-*11*). Along with the well-known inflammatory marker high sensitivity C-reactive protein, Lp-PLA_2_ has gained increasing significance and has been identified as an independent predictor of cardiovascular disease (CVD) in several studies, with elevated Lp-PLA_2_ activities associated with an increased risk of CVD (*7*, *8*, *12*, *13*). Moreover, since half of first cardiovascular events occur in apparently healthy subjects showing normal lipid profiles, Lp-PLA_2_ measurement can be of particular clinical relevance in primary prevention, as it could help identify patients with unrecognized cardiovascular risk when the evaluation of traditional risk markers is not sufficiently informative (*8*, *14*, *15*). A consensus panel issued in 2008 recommended testing Lp-PLA_2_ not only as a diagnostic test for vascular inflammation but also in adjunct to traditional risk factor assessment in individuals with moderate or high risk of cardiovascular disease as defined by Framingham risk scores (*16*). This would allow improved identification of patients at high or very high CVD risk who would benefit from intensification of lipid-modifying therapy, and a lower LDL-cholesterol goal concentration is suggested when Lp-PLA_2_ activities are high (*17*). Different assays for the measurement of Lp-PLA_2_ activity in human plasma and serum have been developed but to date only the PLAC^®^ Test for Lp-PLA_2_ Activity Kit (diaDexus, Inc., San Francisco, USA) has received clearance by the Food and Drug Administration (FDA), in December of 2014.

In the present study we evaluated the analytical characteristics of the PLAC^®^ Test on a Siemens Dimension Vista^®^ 1500 analyzer (Siemens Healthcare Diagnostics Inc., Newark, USA), which is currently installed in our Core Clinical Chemistry Laboratory in an automated configuration. Since reference intervals (RIs) for Lp-PLA_2_ activity in the Italian population have not been reported yet, we analysed serum samples from a cohort of Italian healthy volunteers and evaluated the distribution of Lp-PLA_2_ activity and the correlation of serum Lp-PLA_2_ activity with circulating lipids and age.

## Materials and methods

### Subjects

For the reference population, serum samples were retrospectively selected from stored spare samples obtained from healthy volunteers, which were previously recruited at Tor Vergata University Hospital. We selected spare serum samples from 250 apparently healthy volunteers (123 males, 127 females), median age 37 (18 - 70) years. All subjects had undergone a visit consisting of a medical history, a physical examination, blood pressure measurement, and routine laboratory blood tests (clinical chemistry, haematology, coagulation), and had given informed consent for the collection, storage and/or reuse of blood samples. Exclusion criteria for our study were history and/or signs of CVD, any chronic disease or severe medical condition, alcohol and/or drug abuse, hypertension (systolic blood pressure ≥ 140 mm Hg and/or diastolic blood pressure ≥ 90 mmHg or under antihypertensive medication), total cholesterol > 5.2 mmol/L, triglycerides > 1.8 mmol/L, and use of lipid-lowering agents. Fasting blood samples had been collected into BD Vacutainer^®^ SST™ II Advance Plus serum separator tubes (Becton, Dickinson and Company, Franklin Lanes, USA) following standard procedures. Sample storage conditions were derived from previously published stability studies (*18*, *19*). Briefly, samples were selected on a daily basis after completion of routine laboratory assessments, which were performed within 1 - 2 hours of collection. Selected blood samples were anonymized and either stored at 4 °C and analysed for Lp-PLA_2_ activity within 1 week or aliquoted and stored at - 70 °C for a maximum of 3 months and then analysed after a single freeze-thaw cycle.

### Methods

#### Lp-PLA_2_ enzyme activity

Lp-PLA_2_ activity was determined on an open, user-defined channel on the Siemens Dimension Vista^®^ 1500 automated chemical analyser (Siemens Healthcare Diagnostics Inc., Newark, DE, USA) using the PLAC^®^ Test Activity Kit developed by diaDexus (diaDexus, Inc., San Francisco, CA) for the quantitative determination of Lp-PLA_2_ activity in human plasma and serum. Principle of the test is based on Lp-PLA_2_ hydrolysing the *sn*-2 position of the substrate, 1-myristoyl-2-(4-nitrophenylsuccinyl) phosphatidylcholine, and producing a coloured reaction product, 4-nitrophenol. Lp-PLA_2_ activity was determined by spectrophotometrically monitoring the rate of 4-nitrophenol formation at 405 nm (secondary wavelength 510 nm) for 10 minutes, according to previously published methods (*20*, *21*). A minimum serum sample volume of 200 μL was required. Total reagent volume to fill an empty Siemens Flex^®^ (Siemens Healthcare Diagnostics Inc., Newark, USA) cartridge was 12.0 mL for R1 (buffer) and 3.2 mL for R2 (substrate). Working volumes for a single test were 100 μL for R1 and 25 μL for R2. Under these conditions, 104 tests could be run with each cartridge. A 5-point calibration was performed using a set of five calibrators made with purified recombinant Lp-PLA_2_ protein to generate activities of 0, 50, 100, 250, and 400 U/L, respectively. Calibrators were measured in triplicate. Calibration was verified by measuring high and low activity controls provided with the kit. Lp-PLA_2_ activity was derived from the calibration curve by plotting change in absorbance versus Lp-PLA_2_ activity in U/L.

Serum samples for method validation were provided by diaDexus. These samples had been previously obtained from healthy volunteers and stored at − 70 °C. For the present study, samples were shipped on dry ice and were received frozen.

#### Precision

Assay precision was evaluated according to the EP15-A2 document of the Clinical and Laboratory Standards Institute (CLSI), using 5 different samples (2 controls and 3 serum samples) with known Lp-PLA_2_ activity distributed throughout the calibration range of the assay (*22*). Intra-assay precision was evaluated by running 20 replicates of each sample in a single assay run. Inter-assay precision was evaluated by running 4 replicates of each sample on 5 different days, with a new calibration every day.

#### Sensitivity

Assay sensitivity was evaluated by analysing 4 samples with known low Lp-PLA_2_ activity (range 0 - 10 U/L) in 5 replicates over 5 calibration runs on 5 different days, for a total of 25 replicates for each sample. As specified by the manufacturer, predefined goals for bias and imprecision were respectively ± 5% difference from reference value and a coefficient of variation (CV) < 20% at the limit of quantitation (LoQ).

#### Linearity

Assay linearity was determined by serial recovery studies performed on 3 pairs of different serum samples with high or low Lp-PLA_2_ activity (range: 96 – 328 U/L). In each serum pair, samples were mixed at ratios of 0:100, 10:90, 20:80, 30:70, 40:60, 50:50, 60:40, 70:30, 80:20, 90:10, and 100:0, for a total of 11 activity levels which were measured in duplicate and then compared to expected calculated results.

#### Method comparison

Since there is no gold standard for Lp-PLA_2_ activity to assess assay accuracy, the Bland-Altman plot was used to perform method comparison between the Dimension Vista^®^ 1500 and the analytical platform used by the manufacturer. Lp-PLA_2_ activity was measured in duplicates in a single assay run on 40 blind serum samples spanning an activity range of 5 - 365 U/L, which were provided by diaDexus. Our results were then compared to the activity values previously measured by diaDexus on the same samples using a Beckman Coulter AU400^®^ analyser (Beckman Coulter Inc., Brea, USA).

#### Hook effect

The effect of substrate depletion due to high concentrations of Lp-PLA_2_ protein on the measurement of its enzymatic activity (“hook effect”) was evaluated as follows: purified recombinant Lp-PLA_2_ was diluted into the calibrator matrix to nominal Lp-PLA_2_ activities of 0, 50, 75, 150, 225, 300, 400, 600, 750, 1000, 1250, 1500, and 2000 U/L. These samples were then analysed in duplicate. The concentration of recombinant Lp-PLA_2_ was measured by the Bradford protein assay.

#### Analytical interferences

For interference testing, selected serum samples with Lp-PLA_2_ activity in the range of 96 to 239 U/L were spiked with different levels of potentially interfering endogenous substances. A 6841.5 μmol/L bilirubin stock solution (Sigma-Aldrich Inc, St. Louis, MO, USA) was diluted in 0.1 M NaOH and spiked in serum samples to a concentration of 342.1 μmol/L. A 200 g/L albumin stock solution (Calbiochem, San Diego, CA, USA) was diluted in distilled water and spiked in serum samples to a concentration of 50 g/L. Baseline albumin concentration was also measured in each sample and total albumin (baseline + supplementary) was taken into account for interference evaluation. Haemolysate solution (in-house, 105 g/L haemoglobin) and Intralipid^®^ 20% emulsion (Sigma-Aldrich Inc, St. Louis, USA; 20% = 2.3 mmol/L triglycerides) were ready to use. Serum samples with respectively 1.5 g/L, 1.25 g/L, and 1.0 g/L haemolysate were prepared. Intralipid^®^ emulsion to a final triglycerides concentration of 2.8 mmol/L was added to selected serum samples. For each substance, controls were prepared by spiking serum samples with the corresponding solvent, when appropriate. Bilirubin, albumin and Intralipid^®^ (triglycerides) were measured on the Dimension Vista^®^ 1500 instrument. Haemoglobin (haemolysate) stock solution was measured on the XE-2100 automated haematology analyser (Sysmex, Kobe, Japan).

#### Lp-PLA_2_ enzyme inhibition

A 5 mM stock solution of the Lp-PLA_2_ inhibitor Darapladib was serially diluted in dimethyl sulfoxide (DMSO) in order to obtain 11 dilution levels with inhibitor concentrations ranging from 0.50 nM to 500 nM. Four different serum samples (Lp-PLA_2_ activity range: 97 - 243 U/L) were divided into 232 μL aliquots and 8 μL of each Darapladib dilution were spiked into individual aliquots from all 4 sera, for a total of 44 test samples. Lp-PLA_2_ activity was measured in inhibitor spiked aliquots, un-spiked aliquots, and aliquots spiked with 8 μL of DMSO alone as a control. Measured values were used to fit a dose-response curve for the determination of the *in vitro* half-maximal inhibitory concentration (IC50) of Darapladib.

#### On-board reagent stability

The R1 (buffer) and R2 (substrate) reagents were transferred to the specific Flex^®^ cartridge and stored at 2 - 8 °C in the refrigerated compartment of the Dimension Vista^®^ 1500 instrument for a maximum of 4 weeks. Lp-PLA_2_ activity was measured in triplicate on high and low controls 3 times a week over a window of 28 days, for a total of 72 determinations, always using the same reagent set and calibration curve performed at day 0. Lp-PLA_2_ activity variations of ± 5% from initial value were considered acceptable.

### Statistical analysis

Statistical analysis was performed using MedCalc for Windows, version 13.0 (MedCalc Software, Ostend, Belgium). Method comparison between the Dimension Vista^®^ 1500 and the Beckman AU400^®^ was performed by Bland-Altman plot and Passing-Bablok regression analysis. The Kolmogorov-Smirnov test was used to assess the normality of distribution of Lp-PLA_2_ activity in the whole study population, and in males and females separately. Reference intervals were defined according to CLSI document C28-A3 using the nonparametric method (minimum sample size required for each partition group: N = 120) and central 95^th^ percentile of reference values (*23*). Differences between genders were tested by unpaired t-test. Spearman’s and Pearson’s correlation test was used to test the association between serum Lp-PLA_2_ activity and serum concentrations of total cholesterol, LDL-cholesterol (directly measured), HDL-cholesterol, and triglycerides and the association between serum Lp-PLA_2_ activity and age in the study population. The values P < 0.05 were considered statistically significant.

## Results

Intra-assay variability analysis yielded within–run CVs ranging from 0.6% to 1.4% (Table 1). Inter-assay variability analysis yielded between-run CVs ranging from 0.9% to 2.0% (Table 1).

**Table 1:** Precision evaluation of the lipoprotein-associated phospholipase A2 activity assay on the Dimension Vista analyzer

**Intra-assay imprecision**				**Inter-assay imprecision**		
**Study sample**	**Lp-PLA_2_, U/L**	**%CV**	**Package insert %CV**	**Lp-PLA_2_, U/L**	**%CV**	**Package insert %CV**
**Low Control**	121 (1.5)	1.2	-	121 (2.4)	2.0	-
**High Control**	295 (2.5)	0.8	-	301 (4.1)	1.4	-
**Serum #1**	98 (0.6)	0.6	-	99 (0.9)	0.9	-
**Serum #2**	200 (2.9)	1.4	-	201 (2.8)	1.4	-
**Serum #3**	254 (2.4)	0.9	-	258 (3.0)	1.2	-
**Range**	98 – 295 (0.6 - 2.9)	0.6 - 1.4	1.3 - 1.4	99 – 301 (0.9 – 4.1)	0.9 - 2.0	1.9 - 2.6
Lp-PLA_2_ - lipoprotein-associated phospholipase A2. Lp-PLA_2_ activities are presented as mean (standard deviation). CV – coefficient of variation. For intra-assay imprecision evaluation, 20 replicates of each sample were measured in a single assay run. For inter-assay imprecision evaluation, 4 replicates of each sample were measured on 5 different days. Ranges of intra- and inter-assay %CVs obtained by the manufacturer on a Beckman Coulter AU400 analyzer (package insert) are also shown.						

LoQ was determined as 5.8 U/L with a CV of 9.4%. Linear regression analysis of Lp-PLA_2_ activity values (range: 96 - 328 U/L) from the serial recovery assay samples compared to expected activity values resulted in slopes ranging from 1.009 to 1.013, intercepts ranging from - 1.134 to 3.097 and R^2^ ranging from 0.998 to 0.999. Detailed results are reported in Table 2.

**Table 2:** Linearity evaluation of the lipoprotein-associated phospholipase A2 activity assay on the Dimension Vista analyzer

**Sample pair**	**High activity sample, U/L**	**Low activity sample, U/L**	**Linear regression**			**Package insert**
**Slope**	**Intercept**	**R^2^**
**#1**	299	96	1.01	- 1.13	0.999	Slope: 0.98 - 1.03
**#2**	316	97	1.01	- 0.17	0.999	Intercept: - 6.8 - 10.7
**#3**	328	97	1.01	3.10	0.998	R^2^: 0.995 - 0.999
Three pairs of serum samples with high and low Lp-PLA_2_ activities were used to obtain 11 Lp-PLA_2_ activity levels in a serial recovery assay. Lp-PLA_2_ activity was measured in duplicate in each level and mean values were compared to expected activity values. Linearity results obtained by the manufacturer on a Beckman Coulter AU400 analyzer (package insert) are also shown.						

**Figure 1:**
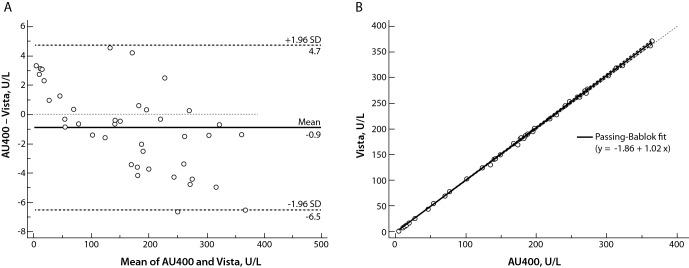
Comparison of lipoprotein-associated phospholipase A2 (Lp-PLA_2_) activity values (N = 40) obtained on VISTA and AU400. (A) In the Bland-Altman plot the solid line shows mean difference, while the dashed lines show the ± 1.96 SD. (B) In the Passing-Bablok regression analysis, the dashed lines show the 95% confidence interval (CI), while dotted line represents identity (X = Y).

The Bland-Altman plot used for the evaluation of agreement between the Dimension Vista^®^ 1500 and the Beckman AU400^®^ (Figure 1A) showed a mean difference of - 0.9 U/L (95% CI: - 1.82 to 0.02), lower limit of - 6.5 U/L (95% CI: - 8.10 to - 4.94), upper limit of 4.7 U/L (95% CI: 3.14 to 6.30). Results of Passing-Bablok regression analysis are also shown (Figure 1B) (Slope = 1.02, 95% CI: 1.01 to 1.03; Intercept = - 1.86, 95% CI: - 3.08 to - 1.26; R^2^ = 0.999).

**Figure 2:**
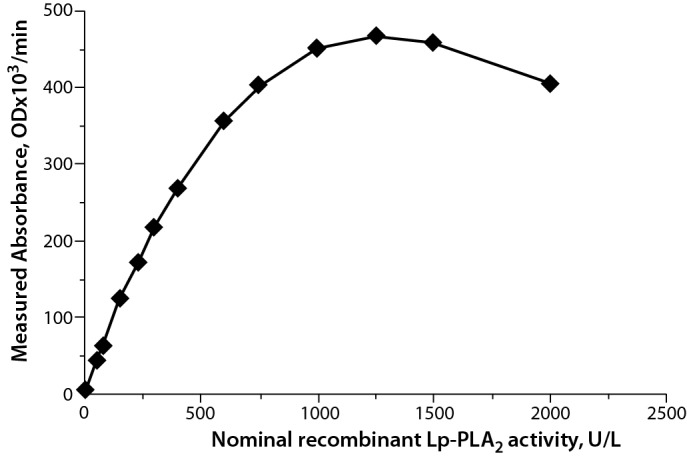
Hook effect evaluation of the lipoprotein-associated phospholipase A2 activity assay on the Dimension Vista analyser. Substrate depletion hooks back measured ODs only at recombinant lipoprotein-associated phospholipase A2 (Lp-PLA_2_) activities > 1000 U/L.

No “hook effect” was observed for measured absorbance up to 450 OD x 10^3^/min, corresponding to a nominal recombinant Lp-PLA_2_ activity of 1000 U/L (Figure 2). Recovery was within 90 - 110%.

Albumin, bilirubin, haemoglobin, and triglycerides (20% Intralipid^®^) were tested as potentially interfering endogenous substances as described in the Methods section. No interference was observed for haemoglobin up to 1.5 g/L, bilirubin up to 342 μmol/L, albumin up to 90 g/L (50 g/L spike + 40 g/L in serum), and triglycerides up to 2.8 mmol/L.

Dose-response data for the determination of the *in vitro* IC50 of Darapladib were obtained by high precision Lp-PLA_2_ activity inhibition assay (0.50 to 500 nM Darapladib spiked into multiple serum aliquots, baseline Lp-PLA_2_ activity range: 97 – 243 U/L). These data were used to fit a dose-response curve to determine the IC50 of the drug. Calculated *in vitro* IC50 of Darapladib was < 50 nM.

Open vial reagents stability on-board the Dimension Vista^®^ was demonstrated for 28 days by mean % difference from initial values of 2.1% (range: − 0.5% to 3.8%) and 0.4% (range: - 3.1% to 2.7%) for low and high activity controls, respectively.

Table 3 shows the main biochemical characteristics of 250 Italian adult healthy volunteers. In this population, Lp-PLA_2_ activity levels presented a Gaussian distribution (P = 0.98) ranging from 71 to 290 U/L, with an average activity of 182 ± 44 U/L (mean ± SD). When separate statistical analysis was performed by gender, a Gaussian distribution was also observed for each gender (males, P = 0.90; females, P = 0.44). Mean Lp-PLA_2_ activity levels in males were significantly higher than in females (203 ± 40 *vs*. 161 ± 37 U/L, respectively; P < 0.001). Lp-PLA_2_ activity distribution amongst population percentiles and reference intervals are summarized in Table 4. In our population, no statistically significant correlation was found between Lp-PLA_2_ activity and LDL-cholesterol (directly measured), HDL-cholesterol, and triglycerides. A moderate positive association was observed between serum Lp-PLA_2_ activity and total cholesterol (r = 0.29, P < 0.001). No statistically significant correlation between Lp-PLA_2_ activity and age.

**Table 3:** Main clinical and laboratory characteristics of the study cohort of healthy donors

**Males, N**	123
**Females, N**	127
**Age, years**	37 (18 - 70)
**Glucose, mmol/L**	4.7 ± 0.5
**Triglycerides, mmol/L**	0.9 ± 0.3
**Total cholesterol, mmol/L**	4.3 ± 0.6
**LDL, mmol/L**	2.8 ± 0.5
**HDL, mmol/L**	1.4 ± 0.3
Age is presented as median (min-max). Results are presented as mean ± SD.	

**Table 4:** Lipoprotein-associated phospholipase A2 activity values in adult healthy subjects

**Percentile**	**Lp-PLA_2_ activity, U/L**		
Entire cohort (N = 250)	Males (N = 123)	Females (N = 127)
**Minimum**	71	87	71
**2.5**	93	107	84
**5**	106	135	97
**20**	144	171	128
**33**	164	187	145
**50**	183	205	165
**67**	203	224	179
**80**	220	241	192
**95**	254	260	217
**97.5**	262	265	225
**Maximum**	290	290	262
**Mean**	182	203	161
**SD**	44	40	37
**2.5-97.5**	93-262	107-265	84-225
Lp-PLA_2_ - lipoprotein-associated phospholipase A2.			

## Discussion

In consideration of the role of Lp-PLA_2_ as a potential marker of vascular inflammation in atherosclerosis, we evaluated the analytical performance of the PLAC^®^ Test for the measurement of Lp-PLA_2_ activity on the Siemens Dimension Vista^®^ 1500 platform, for a possible application in routine clinical chemistry. Performance and practicability of the Dimension Vista^®^ 1500 system are highly suitable for both routine and emergency use high-throughput system. Correlation studies were performed comparing our results obtained using the PLAC^®^ Test for Lp-PLA_2_ Activity Kit on a Siemens Dimension Vista^®^ 1500 instrument to values previously established by the manufacturer using a Beckman Coulter AU400^®^ platform. We show that the PLAC^®^ Test exhibits very good overall analytical performance characteristics on the Dimension Vista^®^ 1500, as revealed by the results obtained for intra- and inter-assay precision, accuracy, linearity and sensitivity. Our results are comparable to the performance characteristics stated in the package insert of the PLAC^®^ Test for Lp-PLA_2_ Activity Kit and to the assay performance established on other platforms and recently published by two independent groups (*18*, *19*).

Substrate depletion experiments demonstrated that the PLAC^®^ Test can be used on a Dimension Vista^®^ analyzer to measure without analytical errors Lp-PLA_2_ activities as high as 1000 U/L. This particular feature would allow the accurate evaluation of patients at high and very high cardiovascular risk, whose Lp-PLA_2_ activities could be more elevated. Analytical interference that may commonly be exerted by high concentrations of some endogenous substances (*i.e*., albumin, haemoglobin, and lipids) was also evaluated and turned out to be very moderate. Moreover, the Dimension Vista^®^ analyzer specifically alerted for potential interference according to standard HIL (haemolysis, icterus, and lipemia) indices. Open-vial stability of the PLAC Test^®^ for Lp-PLA_2_ Activity reagents stored on-board the Dimension Vista^®^ was evaluated by means of repeated measurements of Lp-PLA_2_ activity using the same reagent set, controls, and calibration curve. Reagents were stable for up to 4 weeks in the refrigerated compartment (2 - 8 °C) of the instrument, which is comparable to the stability claims reported by the manufacturer for the PLAC Test^®^ for Lp-PLA_2_ activity on other automated analytical systems. The maximum number of determinations performed with a single reagent cartridge (104 tests) is consistent with many other routine tests already validated for this analyzer.

Recently, Lp-PLA_2_ has been considered as a possible target for the treatment of atherosclerosis, and the selective Lp-PLA_2_ inhibitor Darapladib has been tested as a possible strategy for the prevention and treatment of cardiovascular disease (*24*). However, the clinical trials conducted so far only yielded unsatisfactory results, so there is no evidence yet to support a strategy of targeted Lp-PLA_2_ inhibition (*25*, *26*). We used Darapladib to evaluate inhibitor effect on Lp-PLA_2_ activity in our samples. In agreement with previous findings, our selective inhibition results confirmed that the PLAC^®^ Test allows the specific measurement of Lp-PLA_2_ activity (*24*).

In the present work we also showed the results of preliminary population studies performed on a cohort of 250 Italian apparently healthy volunteers (123 males, 127 females, aged 18–70 years). We found that Lp-PLA_2_ activities presented a Gaussian distribution in the whole population as well as in the two gender groups, with mean Lp-PLA_2_ activity in males significantly higher than in females (P < 0.001) which is in agreement with previously published studies (*27*, *28*). Moreover, this study establishes for the first time RIs of serum LP-PLA_2_ activity in a healthy Italian population and proposes gender-specific RIs (107 - 265 U/L for males and 84 - 225 U/L for females) for clinical practice.

Recent recommendations have been issued for Lp-PLA_2_ activity measurement especially in intermediate cardiovascular risk patients, in order to decide whether they should be moved to a higher risk category (*16*, *17*). However, uniform reporting of clinically relevant cut-points for Lp-PLA_2_ activity in terms of increased risk for cardiovascular events has not been achieved yet. As claimed by the manufacturer, the PLAC^®^ Test incorporates a cut point of 225 U/L for Lp-PLA_2_ activity (which is expressed as nmol/min/mL in the package insert), which would identify patients at increased risk for coronary heart disease (CHD) events. This value was derived from the results obtained by the analysis of plasma samples from 5446 subjects in the placebo arm of the JUPITER Study, by which a cut point of 225 U/L was derived to differentiate subjects at increased risk for CVD events by Cox proportional hazards regression (*29*). Use of the proposed cut-point in our whole study population identifies 84% and 16% of the 250 subjects with low or high activity, respectively. In a population outcome-based sub-study conducted on nearly 5000 subjects from the REGARDS Study, it has been shown that Lp-PLA_2_ activity above the sex-specific 80^th^ percentile (250 U/L in males, 200 U/L in females) was associated with CHD risk over 5.3 years by Cox proportional hazards regression (*30*). These values are very similar to the sex-specific 80^th^ percentiles of our study population (241 U/L in males, 192 U/L in females).

In the healthy donor study cohort, there was no statistically significant association between age and Lp-PLA_2_ activity, while a moderate positive correlation was observed with Lp-PLA_2_ activity and total cholesterol, but not LDL cholesterol. This may well be due to the fact that part of Lp-PLA_2_ is carried by HDL also, while total cholesterol, which reflects the sum of LDL, HDL and, to a lower extent, other cholesterol carrying lipoproteins, is more directly associated with circulating Lp-PLA_2_.

Although our research has reached its aims, this study has some limitations. In particular, the selected population of healthy donors is relatively young (median age 37 years), and therefore our findings may not translate to older populations. Nevertheless, our preliminary data are encouraging, and we are planning to extend this study in the future by increasing the number and age of enrolled participants. We also believe that further analysis of potential endogenous interferents (for example, higher concentrations of triglycerides) as well as selected exogenous agents such as lipid lowering drugs, would be valuable in order to meet suitable analytical conditions for the measurement of Lp-PLA_2_ activity in patients with treated and untreated dyslipidemia.

Based on our results, we can conclude that the PLAC^®^ Test allows accurate and precise measurement of serum Lp-PLA_2_ activity on the Dimension Vista^®^ 1500 automatic analyzer. Our findings add to the results previously obtained on other automatic clinical chemistry analyzers, and can contribute to the method harmonization process for Lp-PLA_2_ activity measurement (*18*, *19*). Taken together, the high-throughput potential of the Dimension Vista^®^ 1500 analyzer and the overall performance characteristics of the PLAC^®^ Test could provide a valuable tool for cardiovascular risk assessment in both primary and secondary prevention of cardiovascular disease, as well as for epidemiological investigations.
